# Combining metformin and esomeprazole is additive in reducing sFlt-1 secretion and decreasing endothelial dysfunction – implications for treating preeclampsia

**DOI:** 10.1371/journal.pone.0188845

**Published:** 2018-02-21

**Authors:** Tu’uhevaha J. Kaitu’u-Lino, Fiona C. Brownfoot, Sally Beard, Ping Cannon, Roxanne Hastie, Tuong V. Nguyen, Natalie K. Binder, Stephen Tong, Natalie J. Hannan

**Affiliations:** Translational Obstetrics Group, The Department of Obstetrics and Gynaecology, University of Melbourne and Mercy Perinatal, Mercy Hospital for Women, Heidelberg, Victoria, Australia; Academic Medical Centre, University of Amsterdam, NETHERLANDS

## Abstract

**Introduction:**

The discovery of new treatments that prevent or treat preeclampsia would be a major advance. Antiangiogenic factors soluble fms-like tyrosine kinase-1 (sFlt-1) and soluble endoglin (sENG) are secreted in excess from the placenta, causing hypertension, endothelial dysfunction, and multiorgan injury. We recently identified metformin and esomeprazole as potential treatments for preeclampsia. Both reduce placental and endothelial secretion of sFlt-1 and soluble endoglin, and reduce endothelial dysfunction.

**Objectives:**

We set out to assess whether combining metformin and esomeprazole would additively reduce sFlt-1 and soluble endoglin secretion and reduce endothelial dysfunction (verses drug alone). Metformin and esomeprazole were added to primary placental cells and tissues, and endothelial cells and their effects on sFlt-1 and soluble endoglin secretion were assessed *in vitro*. Tumor necrosis factor-α (TNF-α) was added to endothelial cells to induce dysfunction *in vitro*. We examined the ability of metformin + esomeprazole to rescue TNF-α induced vascular cell adhesion molecule-1 (VCAM-1) and Endothelin-1 (ET-1) expression, leukocyte adhesion (markers of endothelial dysfunction).

**Results:**

Combining metformin and esomeprazole was additive at reducing sFlt-1 secretion and expression of sFlt-1 e15a mRNA isoform in primary cytotrophoblast, placental explants and endothelial cells. In contrast, no additive reduction in sENG was observed with combined metformin and esomeprazole. The low-dose combination of metformin + esomeprazole additively reduced TNF-α-induced VCAM-1 mRNA, but not VCAM-1 protein expression. There was no additive reduction when combining metformin and esomeprazole on TNF-α induced PBMC adhesion to endothelial cells. However, combining metformin and esomeprazole additively reduced ET-1 mRNA expression.

**Conclusions:**

In conclusion combining metformin and esomeprazole additively reduced secretion of sFlt-1, and markers of endothelial dysfunction. The combination of metformin and esomeprazole may provide a more effective treatment or prevention for preeclampsia compared to either as single agents.

## Introduction

Preeclampsia is globally responsible for tens of thousands of maternal and neonatal deaths each year[1, 2]. Currently, there are no medical therapies to halt disease progression and expectant management and delivery remains the mainstay of treatment[1, 3–6].

An important step in the pathogenesis of preeclampsia is poor placental invasion[7, 8] and the subsequent release of the anti-angiogenic factors soluble fms-like tyrosine kinase 1 (sFlt-1)[9–12] and soluble endoglin (sEng)[13] into the maternal circulation[8, 14–16]. sFlt-1 binds to, and antagonizes pro-angiogenic factors such as vascular endothelial growth factor and placental growth factor whilst sEng antagonizes Transforming Growth Factor β. Together these anti-angiogenic effects result in widespread maternal endothelial dysfunction leading to the multisystem organ injury that is observed clinically[3, 17–19]. Identifying a medical treatment safe in pregnancy and able to quench the release of sFlt-1 and sEng as well as reduce endothelial dysfunction may provide an approach to treat, or prevent this disease.

We have recently reported the possibility that esomeprazole and metformin have potential to treat, or prevent preeclampsia[20, 21]. Importantly, both appear to have a good safety profile in pregnancy[22, 23].

Esomeprazole is a proton pump inhibitor (PPI), a class of drugs widely prescribed to relieve gastric reflux. Importantly, large epidemiology studies have demonstrated PPIs are safe in pregnancy, even if administered during the first trimester[22, 24]. We recently published evidence to show PPIs offer potential candidates to treat preeclampsia[21]. PPIs potently decrease sFlt-1 and sEng secretion from placental cells and tissues, and primary endothelial cells. Endothelial dysfunction, vasoconstriction and hypertension are hallmarks of preeclampsia and our *in vitro and ex vivo* evidence indicated that PPIs rescued many aspects of endothelial dysfunction. We showed that PPIs reduced TNF-α-induced vascular cell adhesion molecule-1 (VCAM-1) and reduced expression of the endothelial derived vasoconstrictor endothelin-1 (ET-1). Importantly, we also demonstrated PPIs are vasoactive *in vivo*, demonstrating their ability to reduce hypertension in a mouse model of preeclampsia[21, 25]. Together this work suggests PPIs may be a therapeutic option for preeclampsia. We are currently testing this in a randomised control Phase II clinical trial (PACTR201504000771349) [26].

The second drug of interest as a potential therapy for preeclampsia is metformin, a drug used to reduce hyperglycaemia in diabetic patients, including women with gestational diabetes[23, 27, 28]. Our pre-clinical analyses of metformin demonstrated it significantly reduced secretion of sFlt-1 from primary human placental cells and tissue as well as from endothelial cells[20]. In addition, we demonstrated metformin reduced endothelial dysfunction, significantly reducing TNF-α-induced VCAM-1 expression and enhancing whole-blood vessel vasodilation[20]. There is also strong clinical evidence to support the potential for metformin to treat preeclampsia. One trial showed as a secondary outcome that the incidence of preeclampsia was reduced in obese women taking metformin from 11 to 3%, while a second meta-analysis demonstrated also via secondary outcome, that metformin reduces pregnancy induced hypertension[29, 30].

A strategy commonly used in drug discovery and clinical care is to combine therapeutic agents, to enhance clinical efficacy and/or to reduce the dose required in order to minimize possible side effects. In regards to clinical efficacy, it would be advantageous if drugs were additive in their actions, but particularly useful if they were synergistic. Given metformin and esomeprazole successfully mitigate key pathogenic features of preeclampsia,[20, 21] we investigated whether combining low-doses of metformin and esomeprazole may be additive or synergistic (or neither) in reducing sFlt-1 and sEng secretion, and mitigating endothelial dysfunction, compared to either drug alone. Thus determining if together lower overall doses could be administered to gain the same result.

## Materials and methods

A full version of the Materials and Methods are included in S1 File.

We performed preclinical studies to examine whether combining metformin and esomeprazole could additively reduce sFlt-1 and sEng secretion from primary placental and endothelial tissues/cells, and whether they are additive in reducing endothelial dysfunction (using various assays of endothelial dysfunction). We performed all functional studies on primary human tissues.

To examine sFlt-1 and sEng secretion, we used primary cytotrophoblast, placental explants (from n = 3 placentas) and human umbilical vein endothelial cells (HUVECs) (from n = 5 umbilical cords). They were treated with metformin or esomeprazole alone, or in combination. sFlt-1 and soluble endoglin were measured via ELISA (RnD Systems). sFlt-1 variant mRNA expression was determined by quantitative RT-PCR.

Endothelial dysfunction was induced in primary endothelial cells (HUVECs) by administering tumor necrosis factor- α (TNF-α). Endothelial cells were treated with metformin, esomeprazole or combination metformin and esomeprazole and the effects on VCAM-1 and Endothelin-1 (ET-1) mRNA determined via quantitative RT-PCR. VCAM-1 protein expression was assessed via western blot and ET-1 secretion measured via ELISA (RnD Systems). Finally the effect of combination treatment on TNF-α- induced peripheral blood mononuclear cell (PBMC) adhesion to endothelial cells was measured.

This study was approved by The Mercy Health Human Research Ethics Committee (R11/34). All participants provided written informed consent. All experiments were repeated at least three times, and biological replicates represent samples obtained from different participants.

## Results

### Combining metformin and esomeprazole additively reduced sFlt-1 secretion from placental and endothelial cells/tissues

Elevated sFlt-1 secretion into the maternal circulation is likely to make a significant contribution to the maternal endothelial dysfunction and hypertension that occurs in preeclampsia[9, 10, 13]. While the major source of sFlt-1 in preeclampsia is the placenta, another source is endothelial cells. We assessed whether combining metformin and esomeprazole were additive at reducing sFlt-1 secretion from primary cytotrophoblasts (isolated from placenta), placental explants and primary endothelial cells (HUVECs).

**Fig 1:**
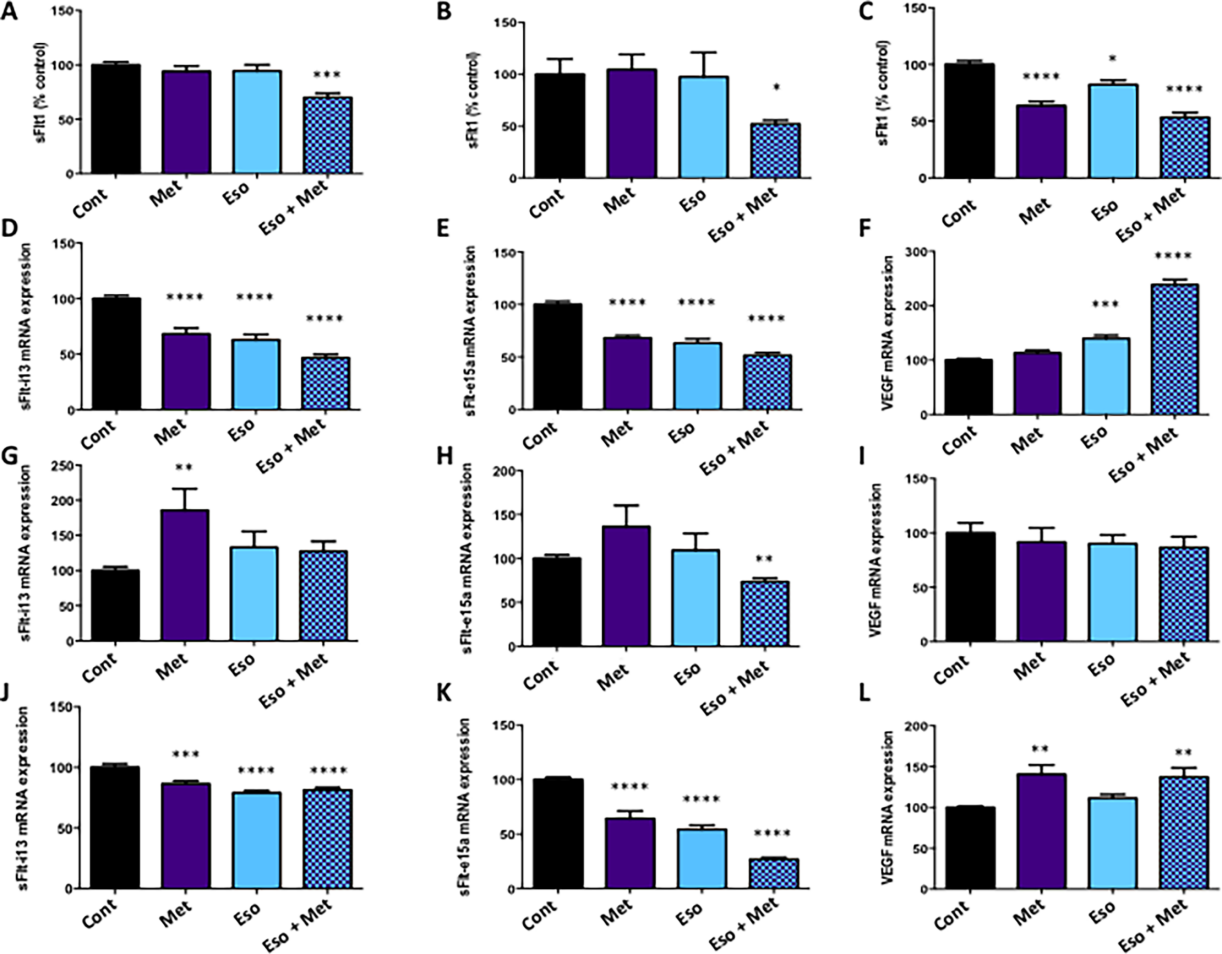
Combining metformin and esomeprazole additively decreases sFlt-1 secretion and expression, and increases VEGF expression in placental and endothelial cells. Isolated primary cytotrophoblast were treated with either metformin alone (Met), esomeprazole alone (Eso) or Met + Eso in combination and the effect on sFlt-1 secretion assessed (A). Neither Met or Eso alone reduced sFlt-1 secretion, however a significant reduction was observed when the two were added in combination (A). Similarly, neither Met nor Eso alone altered sFlt-1 secretion from placental explants, but sFlt-1 secretion was significantly reduced with combination Met and Eso treatment (B). In primary HUVECs, secretion of sFlt-1 was significantly reduced with all treatments compared to control (C). In primary cytotrophoblast both Met and Eso alone significantly reduced sFlt-1 i13 (D) and sFlt-1 e15a mRNA expression (E). A significant increase was observed in VEGF mRNA expression with Eso and Met treatment (F). Met treatment of placental explants caused a significant increase in the expression of sFlt-1-i13 (G) whilst combination Met + Eso caused a significant reduction in the expression of sFlt-1-e15a (H). No change was observed in placental VEGF mRNA expression with any treatment (I). In primary HUVECs, significant reductions in sFlt-1 i13 (J) and sFlt-1 e15a (K) compared to control were observed when Met or Eso were added alone. Combination Met + Eso also significantly reduced expression of both variants compared to control (J-K). VEGF mRNA was significantly increased with either Met alone, and with combination of Met + Eso treatment (L). The following treatment doses were used: *Cytotrophoblasts*: Met; 125 μM, Eso; 25 μM or same doses in combination. *Placental explant tissue*: Met; 500 μM, Eso; 25 μM or same doses in combination. *HUVECs*: Met; 1000 μM, Eso; 25 μM or same doses in combination. All treatments were carried out over 48 h. Data are mean fold change from control ± SEM (*p<0.05, **p<0.01, ***p<0.001, ****p<0.0001 compared to control). (n ≥3).

In primary cytotrophoblast, low-dose metformin (125 μM) or esomeprazole (25 μM) alone did not significantly alter sFlt-1 secretion (Fig 1A; see S3 File). We selected these concentrations and noted them as low on the basis of our previous published work, where these doses caused little or no change in sFlt-1 secretion^18,23^. When both drugs were combined at the same concentrations, we observed a significant additive reduction in sFlt-1 secretion (Fig 1A; see S3 File) compared to control (p<0.001) or both drugs alone (p<0.01). Combining the drugs did not affect cell viability, assessed by MTS assay (data not shown).

We next assessed placental explants (Fig 1B; see S3 File). Placental explants represent a heterogeneous mix of cells preserved within the tissue architecture, and include the surface syncytiotrophoblast, underlying cytotrophoblasts, immune cells and stromal tissue. Drug concentrations were again chosen and defined as low on the basis of our previously published work when we assessed them as single agents^18,23^. Treating placental explants with low-dose metformin (500 μM) or esomeprazole (25 μM) alone had no significant effect on sFlt-1 secretion. When the drugs were combined, we observed significantly reduced sFlt-1 secretion (Fig 1B; see S3 File) compared to control (p<0.05) and metformin alone (p<0.05). Therefore, combining low-dose metformin and esomeprazole may be additive in reducing sFlt-1 secretion from primary cytotrophoblast and placental explants.

We next tested combination treatment on primary endothelial cells (Fig 1C; see S3 File). Both metformin and esomeprazole alone significantly reduced sFlt-1 secretion compared to control (p<0.0001 and p<0.05 respectively). Combining metformin and esomeprazole also significantly reduced sFlt-1 secretion (p<0.0001), however this was only significantly different to esomeprazole alone (p<0.001) and not metformin alone (Fig 1C; see S3 File).

### Combining metformin and esomeprazole reduces sFlt-1 and increases VEGR transcription

Next, we examined the effect of combining metformin and esomeprazole on the mRNA expression of the two major sFlt-1 variants. sFlt-1 e15a is the predominant variant expressed in human placenta[31, 32] whilst sFlt-1 i13 is the most abundant sFlt-1 variant expressed by endothelial cells[31]. In primary cytotrophoblast, both metformin and esomeprazole alone significantly reduced sFlt-1 i13 and sFlt-1 e15a mRNA expression compared to control (Fig 1D and 1E; see S3 File). Combining metformin and esomeprazole significantly reduced both variants compared to control and this effect was significantly lower than either drug alone, indicating an additive effect (p<0.01 compared to metformin alone and p<0.05 compared to esomeprazole alone for both variants; Fig 1D and 1E; see S3 File). Esomeprazole significantly increased VEGF mRNA expression (Fig 1F; p<0.01), however combining esomeprazole and metformin caused an additive increase in VEGF mRNA expression (Fig 1F; see S3 File p<0.001).

When administered to placental explants (Fig 1F and 1G; see S3 File), surprisingly, metformin significantly increased sFlt-1 i13 mRNA expression, whilst no effect of esomeprazole or combination metformin and esomeprazole was observed (Fig 1G). Although the mRNA expression of the placental variant sFlt-1 e15a was not altered by metformin or esomeprazole alone, it was significantly reduced with combined metformin and esomeprazole treatment compared to control (Fig 1H; see S3 File p<0.01). However, this reduction was not significantly different to either drug alone, suggesting no additive effect. Placental explant VEGF mRNA expression was unchanged by the addition of esomeprazole and metformin (Fig 1H; see S3 File).

Finally we assessed sFlt-1 variant expression in primary HUVECs (Fig 1I and 1J; see S3 File). Both metformin and esomeprazole alone significantly reduced sFlt-1 i13 mRNA expression compared to control, with no additive effects of combination treatment (Fig 1I; see S3 File). Metformin and esomeprazole alone also significantly reduced the mRNA expression of sFlt-1 e15a compared to control and combining the two drugs produced a significant and additive reduction (73% reduction; Fig 1I; p<0.0001 compared to metformin alone and p<0.001 compared to esomeprazole alone; see S3 File).

Therefore, combining metformin and esomeprazole appears to be additive in reducing expression of sFlt-1 e15a in primary cytotrophoblast and primary HUVECs.

### Combining metformin and esomeprazole is not additive at reducing sEng secretion

We have previously shown both metformin[20] and esomeprazole[21] reduce sEng secretion. Here, we tested lower doses of metformin and esomeprazole in combination on placental explants and primary HUVECs. sEng secretion from primary cytotrophoblasts was not measured as we have previously shown they secrete very low levels of sEng [33].

**Fig 2:**
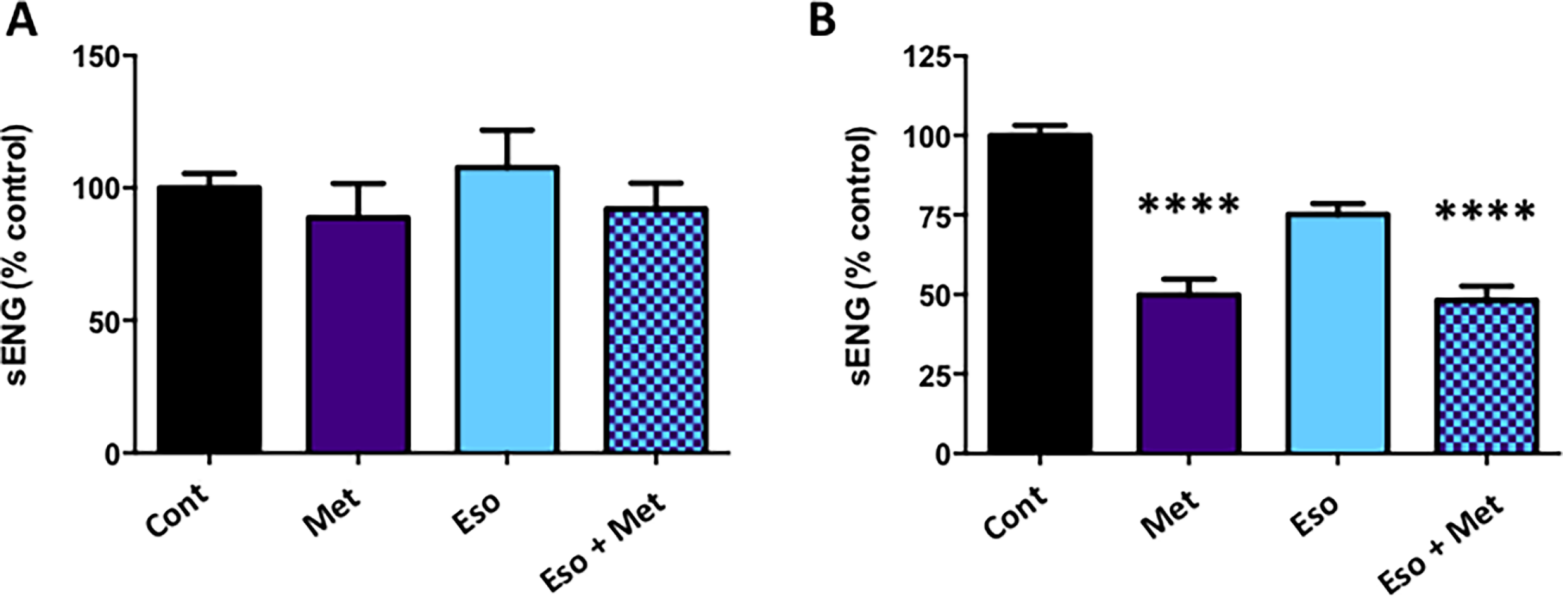
Combination of metformin and esomeprazole are not additive in reducing sEng secretion. Levels of secreted sEng were measured in media collected from placental explant tissue (A) and human umbilical vein endothelial cells (HUVECs) (B), treated with either metformin (Met), esomeprazole (Eso) or Met + Eso in combination. No effect on placental explant secretion of sEng was observed for the drugs alone or in combination (A). In primary HUVECs, Met alone, and Met + Eso caused a significant reduction in sEng secretion from HUVECs compared to control (B). The following treatment doses were used: *Placental explant tissue*: Met; 500 μM, Eso; 25 μM or same doses in combination. *HUVECs*: Met; 1000 μM, Eso; 25 μM or same doses in combination. All treatments were carried out over 48 h. Data are mean fold change from control ± SEM (****p<0.0001 compared to control). (n ≥3).

sEng secretion was not altered by metformin or esomeprazole alone in placental explants at the concentrations examined, nor was it affected by adding the drugs in combination (Fig 2A; see S3 File). In contrast, when adding these drugs to HUVECs, metformin alone significantly reduced sEng secretion, whilst esomeprazole induced a non-significant reduction (Fig 2B; see S3 File). Combining metformin and esomeprazole significantly reduced sEng secretion from HUVECs compared to control, but was not additive (p<0.0001 compared to esomeprazole, not significant compared to metformin alone).

Thus, low dose metformin and esomeprazole do not appear to additively reduce sEng secretion from placental explants, or primary HUVECs.

### Combining metformin and esomeprazole is additive in reducing endothelial dysfunction

Given endothelial dysfunction plays a significant role in preeclampsia, we examined the effects of low dose combination metformin and esomeprazole on endothelial dysfunction using several established *in vitro* models[20, 21, 34–36].

**Fig 3:**
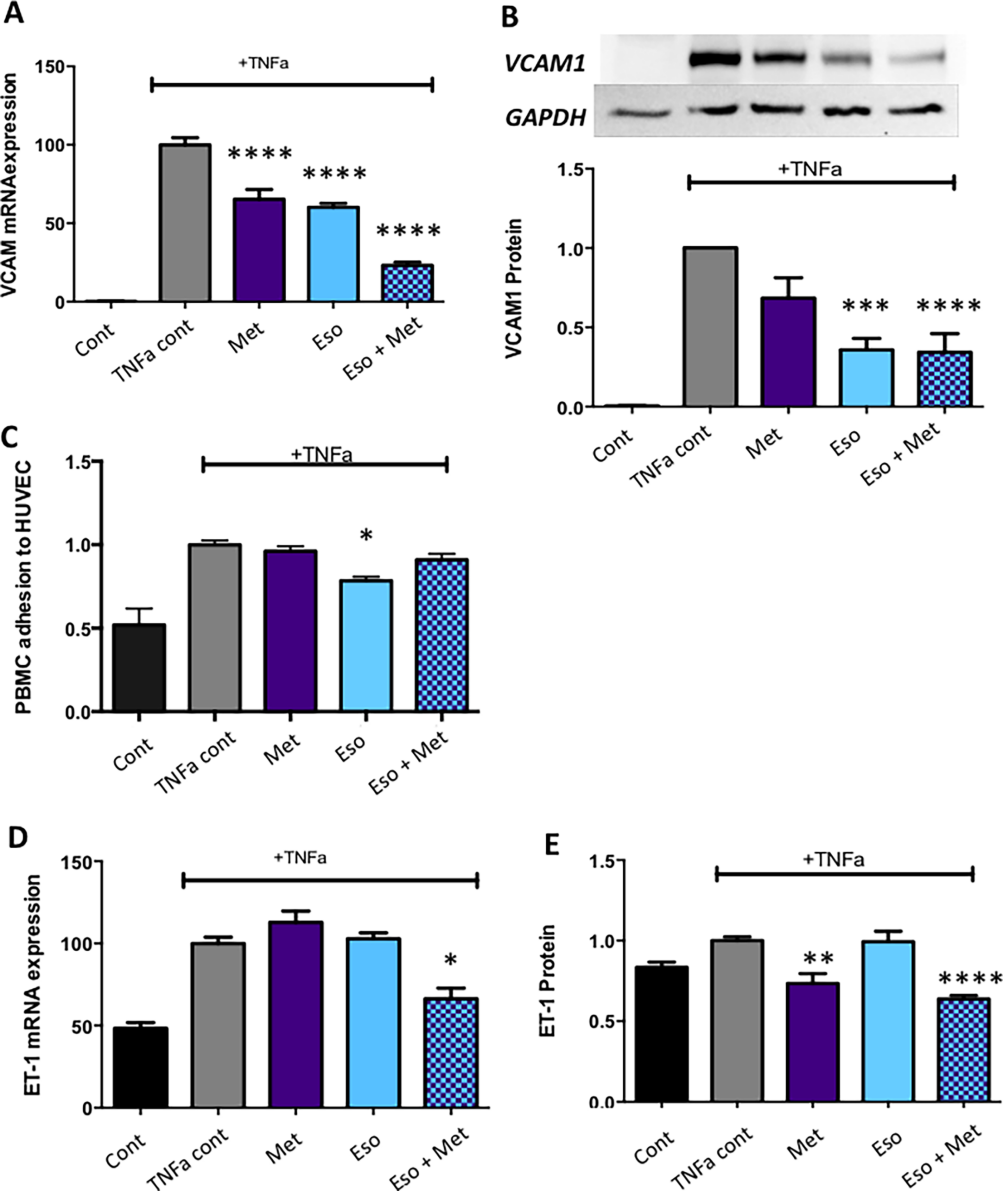
Combination of metformin and esomeprazole additively reduce endothelial dysfunction. Both mRNA (A) and protein (B) expression of Vascular cell adhesion molecule-1 (VCAM) is potently increased in HUVECs with TNF-α. Metformin (Met) and Esomeprazole (Eso) alone and combination Met + Eso significantly reduced VCAM-1 mRNA expression compared to TNF-α alone (A). Combination Met + Eso was more effective at reducing VCAM-1 mRNA than either drug alone (p<0.0001 compared to Eso alone and Met alone). Eso alone and combination Met + Eso significantly reduced VCAM-1 protein expression compared to TNF-α alone (B). Monocyte adhesion to HUVECs is enhanced following TNF-α treatment (C). Monocyte adherence was significantly reduced with Eso treatment, but Met alone or the combination of Met + Eso did not alter monocyte adherence to TNF-α treated HUVECs (C). ET-1 is also increased in HUVECs following treatment with TNF-α treatment (D, E). Whilst neither drug alone significantly altered ET-1 mRNA (D) combination Met + Eso significantly reduced ET-1 mRNA expression compared to TNF-α alone. Both Met alone and combination Met + Eso significantly reduced ET-1 protein compared to TNF-α alone (E). TNF-α was administered for 2 h prior to drug or vehicle treatment, TNF-α was present throughout drug treatment (over 24 h). Met was administered at 1000 μM, esomeprazole at 25 μM or both in combination at the same doses. Data are mean fold change from TNF-α control ± SEM (*p<0.05, ***p<0.001, ****p<0.0001). (n ≥3).

TNF-α was used to induce endothelial dysfunction in primary HUVECs and the expression of vascular cell adhesion molecule (VCAM-1; a marker of endothelial dysfunction)[37] assessed. In the presence of 10ng/ml TNF-α, VCAM-1 mRNA expression was significantly increased compared to cells with no TNF-α (Fig 3A; see S3 File). Esomeprazole alone (25 μM), metformin alone (1000μM) and combination metformin and esomeprazole significantly reduced VCAM-1 mRNA expression compared to TNF-α alone (Fig 3A). The combination treatment was additive, reducing VCAM-1 mRNA expression by 80% (p<0.0001 compared to esomeprazole or metformin alone; Fig 3A). We also examined VCAM-1 protein expression (Fig 3B; see S2 File and S3 File). Compared to TNF-α alone, a significant reduction in VCAM-1 protein expression was observed for esomeprazole alone, and combination metformin and esomeprazole (Fig 3B; see S3 File), however no additive reduction was observed at the protein level.

Given VCAM-1 is an adhesion molecule that may cause enhanced leukocyte tethering in dysfunctional vessels, we next assessed whether combining metformin and esomeprazole would reduce peripheral blood mononuclear cell (PBMC, white cells which we isolated from pregnant women) adhesion to TNF-α treated HUVECs (Fig 3C; see S3 File). Esomeprazole significantly reduced PBMC adhesion in the presence of TNF-α (compared to TNF-α alone), however no effect of metformin alone, or combining metformin and esomeprazole was observed (Fig 3C; see S3 File). Together this data suggests that metformin and esomeprazole may be additive in reducing cell adhesion molecule expression (such as VCAM-1), but that this reduction does not translate to a significant reduction in leukocyte adhesion.

Endothelin-1 (ET-1) is a potent vasoconstrictor elevated in preeclampsia. TNF-α induced expression of ET-1 mRNA expression, however no significant effect of either low-dose metformin or esomeprazole alone was observed on ET-1 mRNA expression. A significant and additive reduction (compared to TNF-α alone) was observed when metformin and esomeprazole were combined (Fig 3D; see S3 File p<0.05 compared to TNF-α alone, p<0.01 compared to metformin alone and p<0.05 compared to esomeprazole alone). We also measured secreted ET-1. Metformin alone and combination metformin and esomeprazole significantly reduced secreted ET-1 compared to TNF-α alone, however the effect of combined treatment was not additive (Not significant when compared to metformin alone, p<0.0001 compared to esomeprazole alone) (Fig 3E; see S3 File).

In accordance with the PLOS ONE journal’s Data Availability requirements the full data sets are provided in S3 File.

## Discussion

We have previously published data [20, 21] demonstrating the potential for metformin and esomeprazole as new therapeutic candidates. Both are drugs that are safe in pregnancy that can reduce the secretion of anti-angiogenic molecules sFlt-1 and sEng, and improve endothelial dysfunction.

The purpose of this study was to investigate whether combining these drugs may be additive, or synergistic in reducing the secretion of sFlt-1 and sEng, and rescuing endothelial dysfunction in our preclinical human *in vitro* models. We specifically chose lower concentrations that had limited or non-significant effects as lone agents, based on our previous work [20, 21]. Given these two drugs are detectable in the micromolar (μM) (or nanograms per milliliter) range [38–40] we specifically chose to examine these agents in a similar range to enhance the potential of translating these finding to clinical trials. We demonstrate that metformin and esomeprazole are additive in reducing sFlt-1 secretion and transcription and additive at reducing some aspects of endothelial dysfunction. Together this suggests that combination treatment may have potential to be more effective in treating preeclampsia, compared to either alone.

### Metformin and esomeprazole additively reduce sFlt-1 secretion and transcription

The main source of sFlt-1 in preeclampsia is secretion from the placenta. Circulating sFlt-1 is elevated in preeclamptic patients preceding clinical onset of disease and serum levels correlate with disease severity[32, 41–43]. Within the circulation, sFlt-1 binds free vascular endothelial growth factor and free placental growth factor, preventing their binding to the normal Flt-1 receptor (membrane bound), thereby antagonising their positive effects on the maternal endothelium[9] and resulting in widespread endothelial dysfunction. Over-expression of sFlt-1 in pregnant rats results in hypertension and proteinuria, clinical hallmarks of preeclampsia[9]. Given the evidence that sFlt-1 is a key anti-angiogenic factor in the pathogenesis of preeclampsia, reducing its secretion is thought to be of key importance when considering a therapeutic to treat this disease. We have previously reported that both metformin[20] and esomeprazole[21] alone significantly reduce sFlt-1 secretion from both placental cells/tissue and endothelial cells. An important finding of this report is that combining low-doses of metformin and esomeprazole is consistently additive in reducing protein secretion of sFlt-1, as well as mRNA transcription of the placental variant sFlt-1 e15a. These effects were consistently observed across three different types of primary tissues, primary cytotrophoblast, placental explants, and primary endothelial cells. Although the reduction in sFlt-1 was not synergistic, the ability to demonstrate an additive reduction using low-doses of these two drugs, highlights their potential to be used in combination in the clinic. VEGF is a pro-angiogenic factor that is mopped up by excess circulating sFlt-1 in preeclampsia, and so we examined whether these agents in combination could additively increase VEGF mRNA expression. We observed an additive significant increase in VEGF mRNA expression in primary trophoblasts treated with metformin and esomeprazole in combination. HUVECs showed increased VEGF expression but this was not additive compared to metformin alone. Interestingly no change in VEGF expression was seen in placental explant tissue treated with metformin of esomeprazole.

We next examined whether combination treatment would significantly reduce sEng secretion. In contrast to sFlt-1, low-dose metformin or esomeprazole did not reduce sEng secretion from placental tissue and was not additive at reducing secretion from either placental or endothelial cells. Although disappointing, the focus of this work was to test low concentrations of these agents in combination preclinical—therefore it is possible that further dose-finding experiments may identify higher metformin and esomeprazole doses that could also additively reduce sEng secretion.

### Combining metformin and esomeprazole reduces VCAM-1 and ET-1 expression

Endothelial dysfunction in preeclampsia results in the clinical manifestation of the disease including systemic inflammation and hypertension. Therefore, it is of interest to assess whether a combination therapeutic could mitigate key features of endothelial dysfunction. In this study, we used a well-characterised model of TNF-α- induced endothelial dysfunction. Our findings show that combining metformin and esomeprazole additively reduced VCAM-1 mRNA expression, but not protein, and their combination did not reduce leukocyte adhesion.

We also demonstrate that combining metformin and esomeprazole additively reduced vasoconstrictor ET-1 mRNA expression. This is of interest given the significant hypertension associated with preeclampsia. Importantly, we have previously shown that both esomeprazole and metformin alone induce significant vasodilation of human blood vessels, thus strengthening the possibility that combined treatment may be beneficial in reducing the vasoconstrictive characteristic (hypertension) of this disease.

Thus together our data demonstrates that combining metformin and esomeprazole additively reduces sFlt-1 secretion, enhances VEGF mRNA expression and also improves aspects of endothelial dysfunction. Given the safety profile of these drugs, our data arguably warrants consideration to test this combination in clinical trials.

A significant strength of our study is the use of primary human cells and tissues rather than cell lines. Our data demonstrated that metformin and esomeprazole in lower concentration in combination are additive in reducing sFlt-1 (and in placenta sEng) secretion, and reducing endothelial dysfunction. Furthermore these studies were undertaken at both the mRNA and protein level using 3 different primary tissue/cells. We speculate that both these agents are able to do this by a common mechanism and this may involve the placental mitochondria given both metformin and the proton pump inhibitors have been shown to have key actions on mitochondrial function [20, 44–46]. However further studies are needed to determine the precise mechanisms, and whether the additive effects observed can be attributed to more than one molecular pathway.

A limitation of this work however is that we are yet to test this drug combination using an *in vivo* model of preeclampsia where it would be useful to show that the two drugs in combination can improve clinical parameters of the disease including hypertension and proteinuria. Further studies to identify the possibility of these agents acting in combination in animal models are planned before moving in to clinical trials.

### Clinical trials to assess the therapeutic potential of metformin and esomeprazole to treat preeclampsia

Given the promising data demonstrating metformin or esomeprazole mitigate key features of preeclampsia, we have been pursuing these concepts in clinical trials. We are currently undertaking a double blind, randomized, placebo-controlled trial to evaluate the efficacy of esomeprazole to treat early onset preeclampsia (PIE Trial, see reference for the trial protocol)[26]. We are recruiting 120 participants with preeclampsia at 26 to 32 weeks and 6 days gestation and administering esomeprazole, or placebo. The primary outcome of this trial is prolongation of gestation measured from time of enrolment to delivery. We expect to finalise enrolment by mid-2017.

Excitingly, there is already clinical evidence suggesting metformin may reduce the incidence of preeclampsia with a randomized controlled trial of metformin in an overweight obstetric population showing a reduction in preeclampsia from 11% in controls to 3% in the treatment group (OR 0.24 95% CI 0.1–0.61) when assessed as a secondary outcome. In light of this clinical data and our preclinical work[20] we are setting up a second clinical trial to assess whether metformin may treat preeclampsia. However, we are mindful of the fact that by the time preterm preeclampsia presents, the disease pathophysiology is already very advanced. It is possible that our trials will find lone agents may have limited efficacy in controlling preterm preeclampsia. In light of these preclinical findings, we plan to pursue trials of combination treatments if our two clinical trials of the drugs as single agents show modest or no efficacy in our clinical endpoints we are examining.

## Conclusion

We have performed preclinical studies using primary human cells and tissues to show that combining metformin and esomeprazole is additive in reducing sFlt-1 and reducing endothelial dysfunction. These results demonstrate that there may indeed be merit in initiating clinical trials to treat preeclampsia using combination therapy.

## Supporting information

S1 File Full Material and methods. (DOCX)

S2 File Vascular cell adhesion molecule 1 (VCAM) and GAPDH protein expression analysis by western blots (blots from individual n = 5 experiments). HUVECs treated with TNFα had increased VCAM1 expression, this was attenuated with Met, Eso and combination on Met + Eso.
(TIFF)

S3 File Raw data files and statistical analysis used for generation of Figs 1–3. (ZIP)
