# Supplementary material for: Combining metformin and esomeprazole is additive in reducing sFlt-1 secretion and decreasing endothelial dysfunction – implications for treating preeclampsia
Source: PLoS One. 2018 Feb 21;13(2):e0188845. doi: 10.1371/journal.pone.0188845 (PMC5821305; doi:10.1371/journal.pone.0188845)
Supplement: S1 File — (DOCX) [file pone.0188845.s001.docx]

**Supplementary Material**

**Materials and Methods**

**Isolation and treatment of primary human cytotrophoblasts**

This study was approved by The Mercy Health Human Research Ethics Committee and all women gave written informed consent for collecting samples. All methods were performed in accordance with the University of Melbourne and Mercy Health guidelines and regulations. Human cytotrophoblasts were isolated from three individual term (≥37 weeks gestation up to 41 weeks gestation) normal placentas, obtained at caesarean section as previously described[^17^](#_ENREF_17). Primary cytotrophoblasts were cultured in DMEM high Glutamax (Life Technologies) containing 10% FCS and 1% antibiotic-antimycotic (Life Technologies) on fibronectin (10 mg/mL; BD Biosciences, New South Wales, Victoria) coated wells. Cells were plated and allowed to attach over 12-18 h before washing with dPBS (Life Technologies) to remove cell debris. Cells were cultured under 8% O_2_, 5% CO_2_ at 37°C for 48 h following treatment.

## Isolation and treatment of primary human umbilical vein endothelial

## cells (HUVECs)

Umbilical cords were collected from three individual normal term placentas, and the cord vein was infused with 10 ml (1 mg/ml) of collagenase (Worthington, Lakewood, New Jersey) and cells isolated as previously described[^18^](#_ENREF_18). Cells were used between passage 2 to 4 and cultured at 37^o^C in 20% O_2_ and 5% CO_2_.

**Esomeprazole and Metformin treatment**

Isolated primary HUVECs were treated with esomeprazole (25 μM), metformin (1000 μM) or both esomeprazole (25 μM) and metformin (1000 μM) in combination and cultured for 48 h. These concentrations (across this time course) were chosen as they demonstrated limited effects on sFlt-1 and sEng secretion in preliminary trials.

For primary cytotrophoblast, concentrations of 25 μM Esomeprazole and 125 μM metformin were chosen. These doses were chosen as they demonstrated limited effects on sFlt-1 secretion in preliminary trials. Primary cytotrophoblast were treated with vehicle control, esomeprazole alone, metformin alone or esomeprazole and metformin in combination for 48 h in culture. At the cessation of treatment, cell viability was assessed using a MTS assay (Promega, Madison, WI), media was collected for ELISA analysis and RNA collected for down-stream analyses.

For whole placental tissue, small pieces of villous tissue were cut from the mid-portion of the placenta from three individual normal term pregnancies. Tissue was thoroughly washed with PBS and allowed to equilibrate (cultured for 24 h without treatment). Explant tissue was then treated with esomeprazole (25 μM), metformin (500 μM) or both esomeprazole (25 μM) and metformin (500 μM) in combination and cultured for further 48 h.  These concentrations were chosen as they demonstrated limited effects on sFlt-1 and sEng secretion in preliminary trials. Media was then collected for ELISA (sFlt-1 and sEng levels after treatment were normalized to tissue weight) and RNA collected for downstream analyses.

**Cell viability assay (MTS assay)**

Cell viability assays were performed using CellTiter 96-Aqueous One solution (Promega, Madison WI) according to the manufacturer’s instructions.

**ELISA Analysis**

Concentrations of sFlt-1 were measured in conditioned cell/tissue culture media using the DuoSet Human VEGF R1/Flt-1 kit (R&D systems by Bioscience, Waterloo, Australia) as per manufacturer's instructions.

For ET-1 analysis, the Human Endothelin-1 Quantikine ELISA kit (R&D Systems) was used according to manufacturer’s instructions.

**Endothelial dysfunction**

Endothelial dysfunction experiments were undertaken using primary HUVECs isolated from five normal term placentas. HUVECs were pre-treated with 10ng/ml Tumor Necrosis Factor-α (TNF-α) (Sigma) for 2h, before doses of esomeprazole (25 μM), metformin (1000 μM) or both esomeprazole (25 μM) and metformin (1000 μM) in combination were added in the presence of TNF-α for a further 24h. At the cessation of the experiment, media was collected for ELISA analysis of endothelin-1 (ET-1) and RNA collected for qRT-PCR measurement of VCAM and ET-1.

*Adhesion Assay*

HUVECs (isolated from three individual normal term placentas) were treated with a constant dose of 10ng/ml TNF-α (Sigma) and with doses of esomeprazole (25 μM), metformin (1000 μM) or both esomeprazole (25 μM) and metformin (1000 μM) in combination for 24 hours at 20% O_2_, 5% CO_2_ and 37°C. For the leukocyte adhesion assay experiment, primary human Peripheral Blood Mononuclear Cells (PBMCs) were isolated from the whole blood of pregnant patients (from three individual healthy women). Blood was collected in an EDTA vacutainer and centrifuged to remove plasma. Red blood cell fraction was diluted with PBS and layered on top of 12ml Ficoll-Paque (GE Healthcare, Little Chalfont, UK). After centrifugation at 400 x g for 30 mins without brakes, the PBMC fraction was collected and washed in PBS to remove excess Ficoll-Paque. Contaminating platelets were removed following a second centrifugation at 300xg for 10 mins. Red blood cells were lysed and PBMCs collected. PBMCs were pre-incubated with calcein (Merck Millipore, Darmstadt, Germany) for 30 minutes at 37°C and applied to HUVECs as previously described[^19^](#_ENREF_19). Fluostar omega fluorescent plate reader (BMG labtech, Victoria, Australia) was used to detect fluorescence (quantify adhesion).

**Quantitative RT-PCR**

RNA was extracted from primary cytotrophoblast and HUVECs using an RNeasy mini kit (Qiagen, Valencia, CA) and quantified using the Nanodrop ND 1000 spectrophotometer (NanoDrop technologies Inc, Wilmington, DE). 0.2 μg of RNA was converted to cDNA using Applied Biosystems high capacity cDNA reverse transcriptase kit (Life Technologies) as per manufacturer guidelines.

Gene expression of *VEGF*, *VCAM-1, ET-1, YWHAZ and GAPDH* (Life Technologies) were quantified by real time PCR (RT-PCR) on the CFX 384 (Bio-Rad, Hercules, CA) using FAM-labeled Taqman universal PCR mastermix and its specific primer/probe set (Life Technologies) with the following run conditions: 50 ^o^C for 2 minutes; 95 ^o^C for 10 minutes, 95 ^o^C for 15 seconds, 60 ^o^C for 1 minute (40 cycles). SYBR RT-PCR was carried out to assess gene expressions of *sFlt-1 e15a* and *sFlt-1 i13*, *YWHAZ* and *GAPDH*. Primers were designed as previously described (Geneworks, South Australia, Australia)[^20^](#_ENREF_20). RT-PCR was performed using the following run conditions: 95 ^o^C for 20 minutes; 95 ^o^C for 0.01 minutes, 60 ^o^C for 20 minutes, 95 ^o^C for 1 minute (39 cycles), melt curve 65 ^o^C to 95 ^o^C at 0.05 ^o^C increments at 0.05 seconds.

**VCAM-1 Western Blot**

20 µg of cellular protein lysates (extracted from five normal term placentas) were separated on 10% polyacrylamide gels with wet transfer to PVDF membranes (Millipore, Billerica, MA). Membranes were blocked prior to incubation overnight with the primary antibody against VCAM1 (Anti-VCAM1 (SC-1504); Santa Cruz Biotechnology, Dallas, Texas at a 1:200 dilution) and GAPDH (Anti-GAPDH 14C10 (2118); Cell Signalling Technology, Danvers, MA at a 1:5000 dilution). Bands were visualized using a chemiluminescence detection system (GE Healthcare Life Sciences) ChemiDoc XRS (BioRad, Hercules, CA). Relative densitometry was determined using QuantityOne software (BioRad). Loading controls were used for densitometric analysis.

## Statistical analysis

Triplicate technical replicates were performed for each experiment, with a minimum of three (up to five) independent biological replicates performed for each *in vitro* study. Data was tested for normal distribution and statistically analyzed as appropriate. When three or more groups were compared a 1-way ANOVA (for parametric data) or Kruskal-Wallis test (for non-parametric data) was used. Post-hoc analysis was carried out using either the Tukey (parametric) or Dunn's test (non-parametric). When two groups were analyzed, either an unpaired t-test (parametric) or a Mann Whitney test (non-parametric) was used. All data is expressed as mean ± SEM. P values <0.05 were considered significant. Statistical analysis was performed using GraphPad Prism 7 software (GraphPad Software, La Jolla, CA).
