# Supplementary figures and images for: Combining metformin and esomeprazole is additive in reducing sFlt-1 secretion and decreasing endothelial dysfunction – implications for treating preeclampsia
Source: PLoS One. 2018 Feb 21;13(2):e0188845. doi: 10.1371/journal.pone.0188845 (PMC5821305; doi:10.1371/journal.pone.0188845)

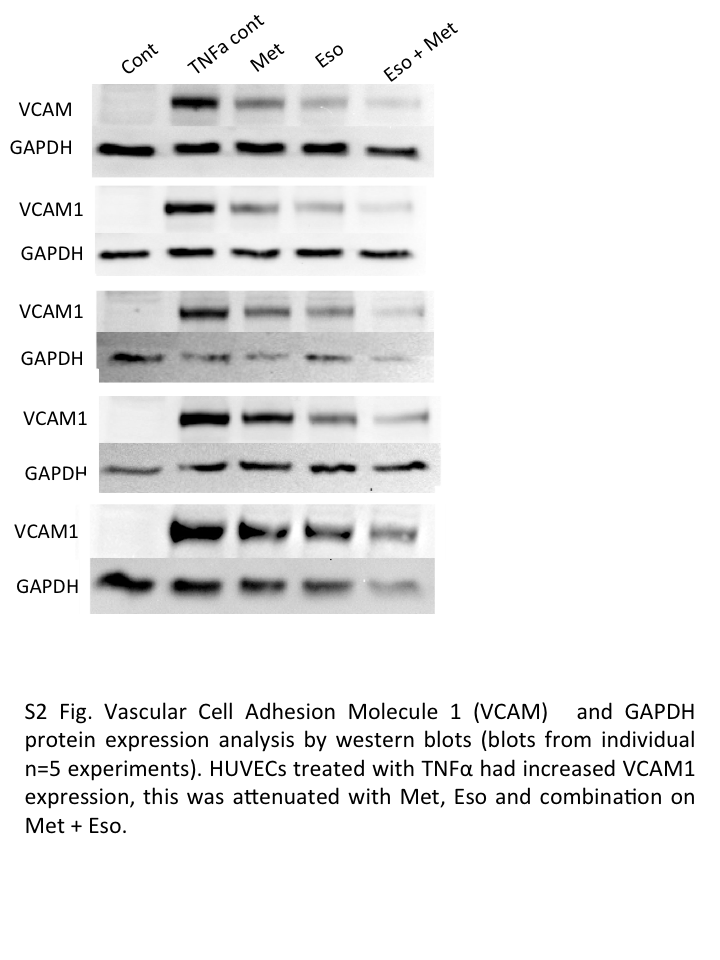

Supplement: S2 File — HUVECs treated with TNFα had increased VCAM1 expression, this was attenuated with Met, Eso and combination on Met + Eso. (TIFF) [file pone.0188845.s002.tiff]
